# Transiently antigen primed B cells can generate multiple subsets of memory cells

**DOI:** 10.1371/journal.pone.0183877

**Published:** 2017-08-29

**Authors:** Jackson S. Turner, Zachary L. Benet, Irina Grigorova

**Affiliations:** Department of Microbiology and Immunology, University of Michigan Medical School, Ann Arbor, Michigan, United States of America; Institut Cochin, FRANCE

## Abstract

Memory B cells are long-lived cells that generate a more vigorous response upon recognition of antigen (Ag) and T cell help than naïve B cells and ensure maintenance of durable humoral immunity. Functionally distinct subsets of murine memory B cells have been identified based on isotype switching of BCRs and surface expression of the co-stimulatory molecule CD80 and co-inhibitory molecule PD-L2. Memory B cells in a subpopulation with low surface expression of CD80 and PD-L2 are predominantly non-isotype switched and can be efficiently recruited into germinal centers (GCs) in secondary responses. In contrast, a CD80 and PD-L2 positive subset arises predominantly from GCs and can quickly differentiate into antibody-secreting plasma cells (PCs). Here we demonstrate that single transient acquisition of Ag by B cells may be sufficient for their long-term participation in GC responses and for development of various memory B cell subsets including CD80 and PD-L2 positive effector-like memory cells that rapidly differentiate into class-switched PCs during recall responses.

## Introduction

Long term humoral immunity is critical for protection from many pathogens and is elicited by most successful vaccines. Upon primary infection or immunization, a small population of Ag-specific B cells becomes activated and expands after acquiring T cell help [1]. Some of these expanded clones differentiate into memory B cells, which circulate and can be rapidly recruited into the humoral immune response upon reacquisition of Ag and T cell help [2,3,4]. Other activated B cells are recruited into GCs, in which they undergo somatic hypermutation of their BCRs and higher affinity clones are selected based on their ability to acquire Ag and T cell help [5]. Memory B cells and antibody-secreting PCs differentiate from GC B cells after iterative rounds of mutation and selection, although memory cells are thought to undergo less stringent affinity-based selection compared to PCs [6,7].

Memory B cells can be most broadly defined as B cells that have been activated by Ag and persist in its absence [8,9]. A number of recent studies have demonstrated that functionally distinct subsets of murine memory B cells can be identified phenotypically, either by expression of an isotype-switched or unswitched BCR [3,4,10] or by expression of combinations of the co-stimulatory and co-inhibitory molecules CD80 and PD-L2 [11,12]. While memory B cells that form prior to GCs are predominantly IgM positive, the majority of memory B cells that differentiate from GCs are class-switched [3,4,13]. Studies employing isotype switching as a marker of functional heterogeneity found that class-switched memory B cells are more “effector memory-like,” that is, they differentiate more rapidly into PCs upon reencountering Ag and T cell help, whereas IgM memory cells were more “naïve-like” in that they were more predisposed to participate in secondary GCs prior to differentiating into PCs [3,10]. Studies using CD80 and PDL2 to differentiate subsets of memory cells identified at least three functionally distinct memory subsets, defined as double negative (DN, CD80^−^PD-L2^−^), single positive (SP, CD80^−^PD-L2^+^), and double positive (DP, CD80^+^PD-L2^+^). Isotype-switched and unswitched memory B cells are present in all three subpopulations, but the DN population consists of predominantly IgM^+^ B cells, whereas the SP and DP populations are comprised of progressively more class-switched cells and demonstrate increasing propensity to differentiate quickly into PCs. While DN cells are the most naïve-like, DP cells are thought to originate predominantly from GCs [11,12,13].

Previously we found that single transient Ag acquisition is sufficient for B cell recruitment into immune responses when T cell help is available, including their participation in histologically-defined GCs and differentiation into PCs and memory B cells [14]. Interestingly, compared to GC and PC responses, the short-term memory B cell response appeared least affected by the dose of transiently acquired Ag or reacquisition of Ag by the participating B cells. However, in that study memory cell subsets and class-switching were not quantitatively assessed. In addition, the memory B cell response was only analyzed out to 21 days, while later timepoints were not examined. Therefore, whether transient acquisition of Ag by B cells is sufficient for their differentiation into the memory B cell subsets described above is not known, and whether memory cells generated by B cells that transiently acquire Ag persist in the periphery is unclear. In the work described below we demonstrate that single transient acquisition of Ag may be sufficient for long-term participation of B cells in GC responses and for development of various memory B cell subsets including DP and class-switched cells that can quickly differentiate into PCs during a recall response.

## Materials and methods

### Mice

C57BL/6 (B6) and Ptprc^a^ Pepc^b^/BoyJ (B6-CD45.1) mice were purchased from the Jackson Laboratory. BCR transgenic Hy10 mice (C57BL/6 background) [15] were generously provided by Jason Cyster. Hy10 mice were crossed with B6-CD45.1 mice and maintained on this background. Donor and recipient mice were 6–12 weeks of age. All mice were maintained in a specific pathogen free environment and protocols were approved by the Institutional Animal Care and Use Committee of the University of Michigan.

### Ag preparation

Duck eggs were locally purchased and duck egg lysozyme (DEL) was purified as previously described [15]. BSA and OVA were purchased from Sigma, and DEL was conjugated to OVA via glutaraldehyde cross-linking as previously described [15].

### Immunization and adoptive transfer

Male recipient mice were immunized s.c. in the flanks and base of tail with 50 μg BSA, OVA, or DEL-OVA emulsified in CFA (Sigma), prepared according to the manufacturer’s directions. Where indicated, recipient mice were reimmunized with 50 μg DEL-OVA emulsified in IFA (Sigma), prepared according to the manufacturer’s directions.

Hy10 B cells were enriched from male and female donor mice by negative selection as previously described [16]. For transient exposure to Ag, purified Hy10 B cells were incubated with DEL-OVA *ex vivo* for 5 minutes at 37°C, washed four times with room temperature DMEM supplemented with 4.5 g/L glucose, L-glutamine and sodium pyruvate, 2% FBS, 10 mM HEPES, 50 IU/mL of penicillin, and 50 μg/mL of streptomycin, and transferred i.v. to recipient mice.

### Flow cytometery

Single-cell suspensions from spleens or draining inguinal lymph nodes (dLNs) were incubated with biotinylated antibodies (S1 Table) for 20 minutes on ice, washed twice with 200 μl PBS supplemented with 2% FBS, 1 mM EDTA, and 0.1% NaN_3_ (FACS buffer), incubated with fluorophore-conjugated antibodies and streptavidin (S1 Table) for 20 minutes on ice, washed twice more with 200 μl FACS buffer, and resuspended in FACS buffer for acquisition. For intracellular staining, surface-stained cells were fixed and permeabilized for 20 minutes on ice with BD Cytofix/Cytoperm buffer, washed twice with 200 μl BD Perm/Wash buffer, incubated with for 20 minutes on ice with fluorophore-conjugated antibodies (S1 Table), followed by two washes with 200 μl Perm/Wash buffer, and resuspended in FACS buffer for acquisition. Data were acquired on a FACSCanto or LSRFortessa and analyzed using FlowJo (TreeStar).

### Statistics

Statistical tests were performed as indicated using Prism 6 (GraphPad). Differences between groups not annotated by an asterisk did not reach statistical significance. No blinding or randomization was performed for animal experiments, and no animals or samples were excluded from analysis.

## Results

**Fig 1:**
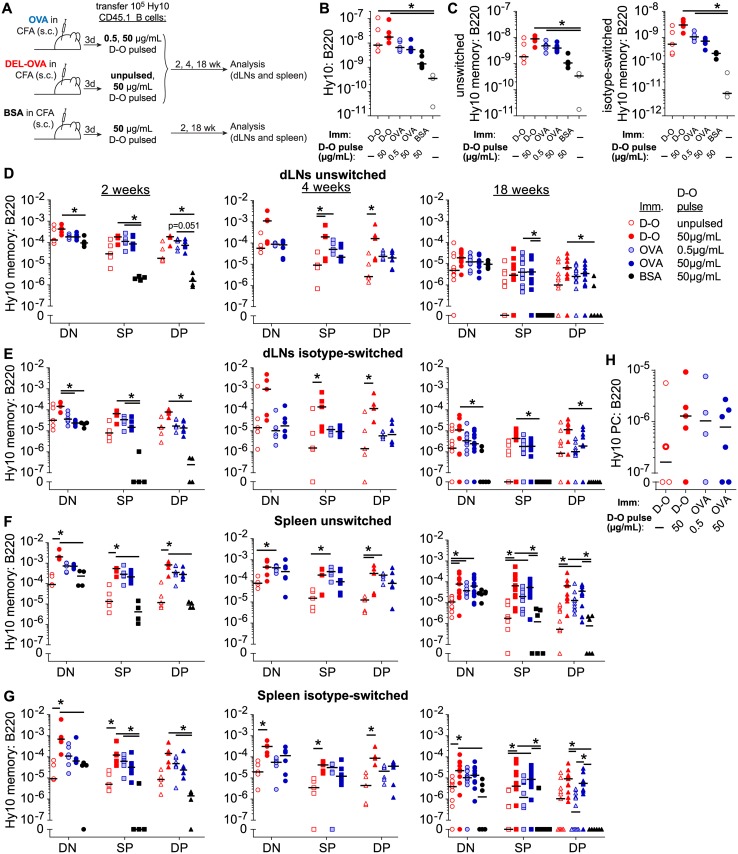
Single acquisition of threshold activating amount of Ag enables generation and persistence of memory B cells *in vivo*. **A**, Experimental outline. Unpulsed Hy10 B cells or Hy10 B cells pulsed *ex vivo* for 5 min with 0.5 or 50 μg/mL DEL-OVA, were transferred into recipient mice s.c. preimmunized with OVA, DEL-OVA, or BSA in CFA. **B**, **C**, Expansion of Hy10 cells in recipient mice 2 weeks after transfer. Total (**B**) and GL7^−^ (**C**) unswitched (left), and isotype-switched (right) Hy10 cells from LNs of recipient mice, shown as fraction of B220 normalized to the number of Hy10 cells transferred. n = 2 independent experiments with 3–6 mice. **D–G**, Memory B cell responses of unpulsed (open symbols) and 50 μg/mL (filled symbols) or 0.5 μg/mL (shaded symbols) DEL-OVA pulsed Hy10 B cells in draining inguinal LNs (dLNs, **D**, **E**) and spleens (**F**, **G**) of OVA (blue symbols), DEL-OVA (red symbols), and BSA (black symbols) immunized recipient mice 2 weeks (left panels), 4 weeks (middle panels) and 18 weeks (right panels) after transfer. DN, SP, and DP subpopulations gated as in S1A and S1C Fig and shown as ratio to total B220^+^CD4^−^CD8^−^ singlet lymphocytes. **H**, Hy10 PC recall response in dLNs 3 days after secondary s.c. immunization with 50 μg DEL-OVA in IFA, 18 weeks after initial transfer of Hy10 cells. For 2 and 4 week timepoints and recall, n = 2 independent experiments with 4–6 mice per condition; for 18 weeks, n = 4 independent experiments, 6–12 mice per condition. Each symbol represents one mouse, line at median. Symbol with thicker line denotes DEL-OVA immunized recipient of naïve Hy10 cells in which unswitched PCs were recovered; all other recovered Hy10 PCs were class-switched. *, P<0.05 (Kruskal-Wallis test with Dunn's post-test between naïve and each immunized condition (**B**, **C**) and all conditions at each timepoint (**D–H**). Differences between groups not annotated by an asterisk did not reach statistical significance.)

To determine the ability of B cells to differentiate into various subpopulations of memory B cells *in vivo* after a single transient acquisition of Ag, and to define how their development and persistence over time depends on the dose of initially acquired Ag and reacquisition of Ag *in vivo*, we used an experimental approach similar to that described before (Fig 1A) [14]. Purified Hy10 B cells specific for avian lysozyme [15,17] were pulsed *ex vivo* for 5 minutes with either a saturating (50 μg/mL) or threshold activating (0.5 μg/mL) concentration of the moderate affinity Ag duck egg lysozyme (DEL) [18] fused to ovalbumin (DEL-OVA) and the unbound Ag was then washed off. 10^5^ Ag-pulsed Hy10 B cells were transferred into recipient mice, which had been s.c. immunized with OVA in CFA three days earlier to activate endogenous OVA-specific helper T cells. Under these conditions, DEL-OVA-primed B cells could not reacquire cognate Ag *in vivo*, but could digest pre-acquired OVA, present OVA-derived peptides, and make cognate interactions with activated OVA-specific Th cells (Fig 1A, upper panel). As a positive control, Hy10 B cells pulsed with a saturating concentration of DEL-OVA as described above or which remained unpulsed were transferred to DEL-OVA immunized mice, in which Ag could be re-acquired (Fig 1A, middle panel). As negative controls, Hy10 B cells were pulsed with a saturating dose of DEL-OVA and transferred to recipient mice preimmunized with the irrelevant Ag BSA. In the BSA-immunized mice no cognate Th cell help would be available for DEL-OVA pulsed Hy10 B cells at the time of their transfer (Fig 1A, lower panel).

We analyzed development of memory B cells by Hy10 cells in the dLNs and spleens of recipient mice at 2, 4, and 18 weeks after B cell transfer. Memory Hy10 B cells were identified as CD4^−^CD8^−^ B220^high^ GL7^low^ CD45.1 cells (S1A Fig). Under all tested conditions the majority of GL7^low^ Hy10 B cells were confirmed to express high levels of CD38 at 2 and 18 weeks, consistent with a memory or naïve B cell phenotype (S1B Fig) [4]. Using the gating strategy outlined in S1C Fig we then identified unswitched (IgM^+^IgD^+/lo^) and class-switched (IgM^−^IgD^−^) GL7^low^ B cells and further subcategorized them into double negative (DN, CD80^−^PD-L2^−^), single positive (SP, CD80^−^PD-L2^+^), and double positive (DP, CD80^+^PD-L2^+^) subsets as described before [11,12]. The gating was defined based on DEL-OVA pulsed Hy10 B cells from the dLNs of DEL-OVA immunized mice relative to naïve Hy10 B cells from unimmunized mice (S1C Fig).

We first examined development of memory B cells by Ag-pulsed Hy10 B cells in the OVA, DEL-OVA and BSA-immunized mice shortly after their transfer (Fig 1A). At two weeks after transfer, Hy10 B cells expanded (Fig 1B) and formed GL7^low^ memory cells in the dLNs of recipient mice under all conditions, exceeding numbers of naïve Hy10 cells recovered from unimmunized recipient mice (Fig 1C). Surprisingly, frequencies of DN class-switched and unswitched GL7^low^ Hy10 cells were almost as high in BSA immunized recipients as in OVA immunized recipients, in which cognate T cell help was available (Fig 1D and 1E, left panels), and the numbers recovered from dLNs were similar (S2A and S2B Fig, left panels). Higher frequencies and numbers of DN switched and unswitched GL7^low^ cells were observed in DEL-OVA immunized recipients in which Hy10 cells pulsed with a large dose of Ag could reacquire Ag *in vivo* (left panels, Fig 1D and 1E and S2A and S2B Fig). Generation of both unswitched and isotype-switched SP and DP memory cells on the other hand, was more similar in dLNs of both DEL-OVA and OVA-immunized mice where T cell help was available, regardless of the amount of Ag B cells initially acquired or their ability to reacquire Ag *in vivo*. At the same time, formation of SP and DP GL7^low^ Hy10 cells was greatly reduced in BSA-immunized control mice (left panels, Fig 1D and 1E and S2A and S2B Fig). Overall, in OVA or DEL-OVA immunized mice SP and DP B cells made up about 30–50% of total Hy10 GL7^low^ cells, (left panels, Fig 1D and 1E and S2A and S2B Fig). A similar pattern of memory cell accumulation was observed in spleens of recipient mice, except for an overall less robust response by unpulsed Hy10 cells transferred to DEL-OVA immunized recipient mice (left panels, Fig 1F and 1G and S2C and S2D Fig). These results suggest that in the presence of T cell help, single transient acquisition of a small amount of Ag by B cells may be sufficient for initial generation of class-switched, SP, and DP subsets of memory B cells.

To determine whether Hy10 memory cells generated in this fashion might have a defect in long-term survival, memory cell persistence was measured at 4 weeks after Hy10 B cell transfer, when resolution of GCs was expected, and 14 weeks later. At 4 weeks a larger population of DP class-switched memory B cells was generated in dLNs and spleens by Hy10 cells that had initially received a large dose of Ag and could reacquire Ag *in vivo* (middle panels, Fig 1E and 1G and S2B and S2D Fig). These results suggest that over time a larger dose of acquired Ag promotes accumulation of effector-like memory cells. However, in the dLNs of OVA- and DEL-OVA-immunized mice, differences in memory subpopulations among these conditions declined substantially by 18 weeks (right panels, Fig 1D and 1E and S2A, S2B and S1D Figs). Of note, while DN Hy10 memory cells were detected in BSA immunized recipients at this time, very few SP or DP memory cells persisted in these mice. In the spleen, a larger population of class-switched DP memory cells was observed in DEL-OVA and OVA immunized mice that received B cells pulsed with a saturating compared to threshold dose of Ag (right panels, Fig 1G and S2D Fig). Consistent with the similar populations of local memory cells among conditions in the dLNs of DEL-OVA and OVA-immunized mice, we found no substantial difference among these conditions in the early class-switched PC recall response in dLNs, which has been shown to be predominantly mounted by class-switched and DP memory B cells (Fig 1H and S1E Fig) [3,10,12].

The higher frequency of isotype-switched and DP memory cells observed 4 weeks after transfer of DEL-OVA pulsed Hy10 B cells in DEL-OVA immunized mice may be potentially explained by prolonged residence in GCs by Hy10 B cells that could acquire and reacquire more Ag *in vivo*. However, less prominent differences in the numbers of isotype-switched DP cells observed at 18 weeks suggest that other factors may have contributed to accumulation or persistence of these memory B cells at later times.

**Fig 2:**
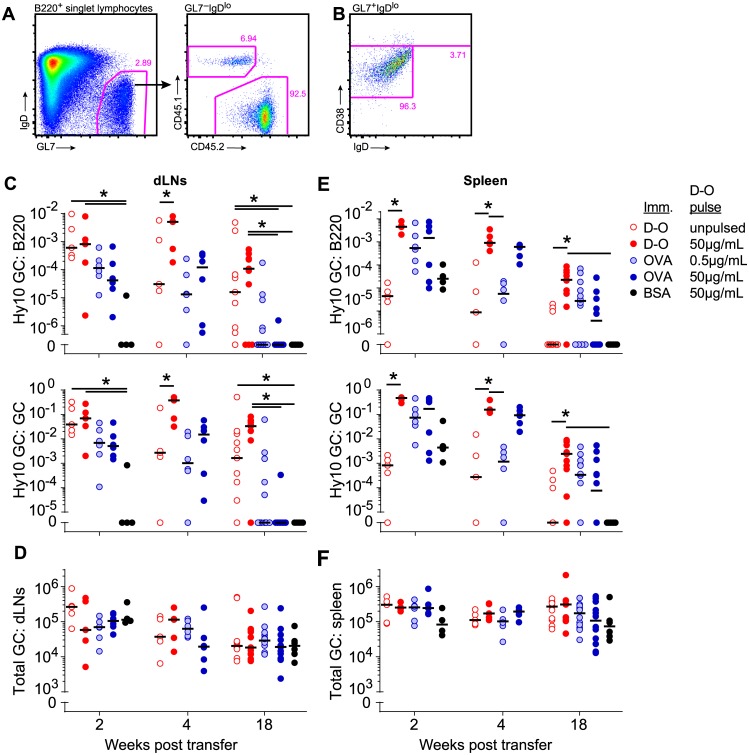
Single acquisition of threshold activating amount of Ag enables GC persistence in spleen. **A**, Hy10 GC B cell gating strategy. See Fig 1A for experimental outline. **B**, CD38 staining of GL7^+^IgD^lo^ B cells in dLNs 2 weeks after Hy10 transfer to recipient mice. Representative of n = 2 independent experiments with 4–6 mice at 2 and 18 weeks after transfer. **C–F**, Hy10 (**C**, **E**) and total (**D**, **F**) GC B cells following transfer of unpulsed (open symbols) and 50 μg/mL (filled symbols) or 0.5 μg/mL (shaded symbols) DEL-OVA pulsed Hy10 B cells in dLNs (**C**, **D**) and spleens (**E**, **F**) of OVA (blue symbols), DEL-OVA (red symbols), or BSA (black symbols) immunized recipient mice at the indicated times post transfer, shown as fraction of total B220^+^CD4^−^CD8^−^ singlet lymphocytes (**C**, **E**, upper panels), fraction of total GC B cells (**C**, **E**, lower panels), or total number of GC B cells per tissue (**D**, **F**). For 2 and 4 week timepoints, n = 2 independent experiments with 4–6 mice per condition; for 18 weeks, n = 4 independent experiments, 6–12 mice per condition. Each symbol represents one mouse, line at median. *, P<0.05 (Kruskal-Wallis test with Dunn's post-test between all conditions at each timepoint. Differences between groups not annotated by an asterisk did not reach statistical significance).

We therefore decided to verify duration of Hy10 B cells’ persistence in GCs under all conditions. The expectations were first, that the GC response would take place in the Ag-draining LNs but not in the spleen; second, that Hy10 B cells pulsed with a threshold dose of Ag would be more rapidly outcompeted from GCs than B cells pulsed with a larger dose of Ag; third, that Hy10 cells that acquired Ag a single time would be more rapidly outcompeted than B cells that could recurrently acquire Ag *in vivo*; and fourth, that Hy10 GC B cells would exit GCs by 4 weeks following their transfer. GC B cells were defined as B220^high^ GL7^high^ IgD^low^ (Fig 2A) and confirmed as CD38^low^ 2 and 18 weeks after transfer (Fig 2B). As previously observed, Hy10 cells were recruited into GCs in dLNs following single or recurrent Ag acquisition in DEL-OVA and OVA, but not BSA-immunized mice (2 weeks, Fig 2C and 2D and S2E Fig) [14]. As expected, 2 weeks after transfer both naïve and Ag-pulsed Hy10 B cells exhibited a trend for more robust GC participation in dLNs of DEL-OVA immunized mice than Ag-pulsed Hy10 B cells in OVA-immunized mice (2 weeks, Fig 2C and S2E Fig). However, similar GC participation was observed by Hy10 cells that transiently acquired a threshold activating dose of Ag compared to cells pulsed with a saturating dose (2 weeks, Fig 2C and S2E Fig). Unexpectedly, Hy10 cells that acquired a single saturating or threshold dose of Ag were able to persist for at least 4 weeks in GCs in OVA-immunized mice, while Hy10 cells transferred into most DEL-OVA immunized mice persisted through the course of the experiment in the dLNs (Fig 2C, S2E Fig).

A GC response by Hy10 B cells in the spleen was even less expected. While naïve unpulsed Hy10 B cells mounted little GC response in the spleens compared to dLNs of mice s.c. immunized with DEL-OVA (Fig 2C–2F and S2E and S2F Fig), we observed robust splenic GC participation by Hy10 B cells pulsed with Ag prior to transfer, regardless of whether recipient mice were immunized with DEL-OVA or OVA, and less robust participation in spleens of BSA immunized recipients. At 4 weeks after transfer GC participation by Hy10 B cells pulsed with a threshold dose of Ag decreased ~10–100-fold. However, participation of Hy10 B cells pulsed with a saturating dose of Ag did not substantially decline at this time, regardless of whether they were transferred into OVA or DEL-OVA immunized mice (4 weeks, Fig 2E and S2F Fig). Finally, by 18 weeks, some Hy10 cells pulsed with either a saturating or threshold dose of Ag and transferred to OVA or DEL-OVA immunized mice still persisted in splenic GCs at similar levels, again regardless of whether they could reacquire Ag *in vivo* (18 weeks, Fig 2E and S2F Fig). Therefore, we observed that B cells that transiently acquired only a threshold activating dose of Ag not only got recruited into GC responses in non-Ag draining secondary lymphoid organs, but in some recipients also persisted (albeit at low levels) in GCs for at least 4.5 months.

## Discussion

In this study we sought to determine whether transiently Ag-pulsed B cells could generate class switched and unswitched CD80^−^PDL2^+^ and CD80^+^ PD-L2^+^ functional memory subpopulations, and whether the initial dose of transiently acquired Ag or B cells’ ability to reacquire Ag *in vivo* altered their persistence in GCs and generation or survival of functional subpopulations of memory B cells.

We found that even a small dose of transiently acquired Ag was sufficient to enable generation of class-switched and SP and DP memory cells and that acquisition of more Ag or recurrent exposure to Ag did not lead to a significant increase in early (2 week) memory B cell numbers of all tested subtypes. Efficient generation of SP and DP memory cells was dependent on acquisition of T cell help, as Hy10 B cells pulsed with a saturating dose of Ag and transferred to BSA immunized mice generated substantially lower numbers of these cells. However, at one month post transfer the frequency of class-switched DP memory B cells was significantly higher for B cells that were pulsed with a large dose of Ag and could reacquire Ag *in vivo*. The observed difference at this time mirrors the prolonged participation in GCs of Ag-pulsed Hy10 B cells that could reacquire Ag *in vivo* and were pulsed with a saturating dose of Ag. Interestingly, by 4.5 months after transfer, the frequencies of class-switched and unswitched SP and DP memory Hy10 B cells in dLNs decreased approximately 10–15 fold and were not significantly different among non-control conditions. Consistent with that, similar early class-switched PC responses were observed after secondary immunization. While the cumulatively observed results suggest that survival of memory B cells generated after a single transient Ag acquisition by B cells were not defective compared to B cells that recurrently acquired Ag, unambiguous interpretation of these findings is complicated by participation of Hy10 B cells in GCs for substantially longer than expected.

Hy10 B cells were able to persist in GCs in dLNs for 18 weeks or longer when recipient mice had been immunized with cognate Ag. Of note, similarly low-level long term GC persistence has been previously observed following primary s.c. immunization with CFA [3], as well as after viral infection [19] and secondary immunization [10]. Such persistence has been attributed to long-term preservation of cognate Ags and their continuous availability to GC B cells. Surprisingly, we observed very efficient recruitment and long-term participation of Ag-pulsed (but not naïve) Hy10 B cells into the GC response in spleens of mice s.c. immunized with either cognate Ag (DEL-OVA) or non-cognate Ag (OVA). While the abundance of Hy10 cells with a GC phenotype dropped over time 100–1000-fold, they persisted in the spleens of some recipients for over 4.5 months. Of note, this was observed even when Hy10 B cells were pulsed with a very low, threshold activating dose of DEL-OVA *ex vivo* and was dependent on the presence of activated cognate helper T cells, as similarly long-term participation was not observed in BSA immunized recipient mice. It is possible that more comparable levels of memory cells observed 4.5 months after the transfer of Hy10 cells that acquired saturating or threshold activating amounts of Ag could be due to continuous input from these small but persistent splenic GC B cells.

We hypothesize that Ag-pulsed Hy10 cells, which are largely retained in the spleen for ~24-36h (unpublished observations), may be able to recruit cognate Th cells and populate GCs at this site. Because less Ag drains to the spleen, transferred Hy10 cells pulsed *ex vivo* with Ag may be able to outcompete endogenous B cells here. This is in contrast to dLNs, in which Hy10 cells that can reacquire cognate Ag compete more successfully with endogenous B cells than those that cannot reacquire Ag, regardless of whether they were pulsed with Ag prior to transfer. This suggests that competition with endogenous GC B cells is more robust in dLNs and that reacquisition of Ag is necessary for Hy10 B cells to persist in GCs here. Another possible explanation for prolonged GC participation by Hy10 cells in the spleen could be acquisition of cross-specificity to continuously available environmental or endogenous Ags as B cells continuously mutate their BCRs in GCs. Such a phenomenon may explain previously recorded occurrences of GC B cells that have little to no detectable specificity to the immunizing Ag [20].

In this study we also observed that Ag-pulsed Hy10 B cells transferred into BSA immunized control mice expanded, but differentiated into SP and DP memory cells substantially less efficiently than in recipients in which cognate helper T cells had been preactivated. In these mice Ag-pulsed Hy10 B cells also participated at low levels in splenic GCs, but did not persist in them long-term. The observed partial participation of Ag-pulsed Hy10 B cells in the memory and GC response in BSA-immunized mice may be an outcome of a T-independent response of activated B cells in the inflammatory milieu induced by immunization. Alternatively, foreign Ag presentation by Hy10 B cells drives modest activation of endogenous DEL- and OVA-specific Th cells that could, in turn, support Hy10 B cells’ participation in some but not all aspects of a T-dependent response.

Overall, in this study we demonstrate that when T cell help is available, transient acquisition of even a threshold activating dose of Ag can enable prolonged participation of B cells in the GC response and generation of various subpopulations of memory B cells, including the class-switched and CD80 PD-L2 based SP and DP memory subsets that quickly differentiate into class switched PCs during recall [3,10,12]. Therefore, this work suggests that functional memory cells can be generated following a single Ag acquisition event and may represent a mechanism to promote a wider diversity of Ag-responsive clones to both persistent as well as transiently arising variants of mutating pathogens.

## Supporting information

S1 Fig Gating strategies. **A**, Hy10 memory B cell gating strategy. Representative of n = 2–4 experiments with 4–12 mice per condition. 2 week timepoint shown from dLNs. **B**, CD38 staining of GL7^−^ Hy10 cells in dLNs. Representative of n = 2 independent experiments with 4–6 mice at 2 and 18 weeks after transfer. 2 week timepoint shown. **C**, Class-switching and memory subpopulation gating. For recovery of high numbers of Hy10 naïve and memory cells for memory subpopulation gating, 5x10^6^ unpulsed or 1x10^6^ DEL-OVA pulsed Hy10 B cells were transferred to naïve and DEL-OVA immunized recipient mice, respectively. Draining LNs from DEL-OVA immunized and peripheral LNs from unimmunized recipients were analyzed 2 weeks after transfer. GL7^−^ Hy10 (left, middle panels) and endogenous (right panels) B cells were gated as in **A**. Representative of n = 2 independent experiments with 3–4 mice. **D**, Example of memory subpopulation gating from 18 week timepoint. Representative of n = 4 independent experiments with 6–12 mice. **E**, Plasma cell gating strategy. PCs were identified as B220^lo^ intracellular Ig^hi^ cells (upper panels, red gates) that were larger and stained more brightly for intracellular Ig than B220^+^ cells (middle panels, black gates). Ig^+^B220^+^ cells (grey gates) shown for comparison. Class switched PCs were defined based on intracellular IgM staining (lower panels).
(PDF)

S2 Fig Single acquisition of threshold activating amount of Ag enables generation and persistence of memory B cells *in vivo*. Memory (**A–D**) and GC (**E**, **F**) B cell responses of unpulsed (open symbols) and 50 μg/mL (filled symbols) or 0.5 μg/mL (shaded symbols) DEL-OVA pulsed Hy10 B cells in dLNs (**A**, **B**, **E**) and spleens (**C**, **D**, **F**) of OVA (blue symbols), DEL-OVA (red symbols), and BSA (black symbols) immunized recipient mice 2 weeks (left panels), 4 weeks (middle panels) and 18 weeks (right panels) after transfer. DN, SP, and DP subpopulations gated as in S1A and S1C Fig, and GCs gated as in Fig 2A. All populations shown as total number of cells per dLNs (**A**, **B**, **E**) and spleens (**C**, **D**, **F**). *, P<0.05 (Kruskal-Wallis test with Dunn's post-test between all conditions at each timepoint. Differences between groups not annotated by an asterisk did not reach statistical significance.)
(PDF)

S1 Table List of antibodies used. (PDF)

## References

[1] McHeyzer-Williams LJ, McHeyzer-Williams MG (2005) Antigen-specific memory B cell development. Annual Review of Immunology 23: 487–513. doi: 10.1146/annurev.immunol.23.021704.115732 15771579 10.1146/annurev.immunol.23.021704.115732

[2] Kaji T, Ishige A, Hikida M, Taka J, Hijikata A, Kubo M, et al (2012) Distinct cellular pathways select germline-encoded and somatically mutated antibodies into immunological memory. Journal of Experimental Medicine 209: 2079–2097. doi: 10.1084/jem.20120127 23027924 10.1084/jem.20120127 PMC3478929

[3] Pape KA, Taylor JJ, Maul RW, Gearhart PJ, Jenkins MK (2011) Different B cell populations mediate early and late memory during an endogenous immune response. Science 331: 1203–1207. doi: 10.1126/science.1201730 21310965 10.1126/science.1201730 PMC3993090

[4] Taylor JJ, Pape KA, Jenkins MK (2012) A germinal center-independent pathway generates unswitched memory B cells early in the primary response. Journal of Experimental Medicine 209: 597–606. doi: 10.1084/jem.20111696 22370719 10.1084/jem.20111696 PMC3302224

[5] Victora GD, Nussenzweig MC (2012) Germinal Centers. Annual Review of Immunology 30: 429–457. doi: 10.1146/annurev-immunol-020711-075032 22224772 10.1146/annurev-immunol-020711-075032

[6] Takahashi Y, Ohta H, Takemori T (2001) Fas is required for clonal selection in germinal centers and the subsequent establishment of the memory B cell repertoire. Immunity 14: 181–192. 11239450 10.1016/s1074-7613(01)00100-5

[7] Shinnakasu R, Inoue T, Kometani K, Moriyama S, Adachi Y, Nakayama M, et al (2016) Regulated selection of germinal-center cells into the memory B cell compartment. Nature Immunology 17: 861–869. doi: 10.1038/ni.3460 27158841 10.1038/ni.3460

[8] Good-Jacobson KL, Shlomchik MJ (2010) Plasticity and heterogeneity in the generation of memory B cells and long-lived plasma cells: the influence of germinal center interactions and dynamics. Journal of Immunology 185: 3117–3125. 10.4049/jimmunol.1001155 20814029

[9] Maruyama M, Lam K-P, Rajewsky K (2000) Memory B-cell persistence is independent of persisting immunizing antigen. Nature 407: 636–642. doi: 10.1038/35036600 11034213 10.1038/35036600

[10] Dogan I, Bertocci B, Vilmont V, Delbos F, Megret J, Storck S, et al (2009) Multiple layers of B cell memory with different effector functions. Nature Immunology 10: 1292–1299. doi: 10.1038/ni.1814 19855380 10.1038/ni.1814

[11] Tomayko MM, Steinel NC, Anderson SM, Shlomchik MJ (2010) Cutting edge: Hierarchy of maturity of murine memory B cell subsets. Journal of Immunology 185: 7146–7150. 10.4049/jimmunol.1002163 PMC3133669 21078902

[12] Zuccarino-Catania GV, Sadanand S, Weisel FJ, Tomayko MM, Meng H, Kleinstein SH, et al (2014) CD80 and PD-L2 define functionally distinct memory B cell subsets that are independent of antibody isotype. Nature Immunology 15: 631–637. doi: 10.1038/ni.2914 24880458 10.1038/ni.2914 PMC4105703

[13] Weisel FJ, Zuccarino-Catania GV, Chikina M, Shlomchik MJ (2016) A Temporal Switch in the Germinal Center Determines Differential Output of Memory B and Plasma Cells. Immunity 44: 116–130. doi: 10.1016/j.immuni.2015.12.004 26795247 10.1016/j.immuni.2015.12.004 PMC4724390

[14] Turner JS, Marthi M, Benet ZL, Grigorova I (2017) Transiently antigen-primed B cells return to naive-like state in absence of T-cell help. Nature Communications 8: 15072 doi: 10.1038/ncomms15072 28429719 10.1038/ncomms15072 PMC5413946

[15] Allen CDC, Okada T, Tang HL, Cyster JG (2007) Imaging of germinal center selection events during affinity maturation. Science 315: 528–531. doi: 10.1126/science.1136736 17185562 10.1126/science.1136736

[16] Allen CDC, Ansel KM, Low C, Lesley R, Tamamura H, Fujii N, et al (2004) Germinal center dark and light zone organization is mediated by CXCR4 and CXCR5. Nature Immunology 5: 943–952. doi: 10.1038/ni1100 15300245 10.1038/ni1100

[17] Goodnow CC, Crosbie J, Adelstein S, Lavoie TB, Smith-Gill SJ, Brink RA, et al (1988) Altered immunoglobulin expression and functional silencing of self-reactive B lymphocytes in transgenic mice. Nature 334: 676–682. doi: 10.1038/334676a0 3261841 10.1038/334676a0

[18] Lavoie TB, Drohan WN, Smith-Gill SJ (1992) Experimental analysis by site-directed mutagenesis of somatic mutation effects on affinity and fine specificity in antibodies specific for lysozyme. Journal of Immunology 148: 503–513. 1729369

[19] Onodera T, Takahashi Y, Yokoi Y, Ato M, Kodama Y, Hachimura S, et al (2012) Memory B cells in the lung participate in protective humoral immune responses to pulmonary influenza virus reinfection. Proceedings of the National Academy of Sciences of the United States of America 109: 2485–2490. doi: 10.1073/pnas.1115369109 22308386 10.1073/pnas.1115369109 PMC3289300

[20] Kuraoka M, Schmidt AG, Nojima T, Feng F, Watanabe A, Kitamura D, et al (2016) Complex antigens drive permissive clonal selection in germinal centers. Immunity 44: 542–552. doi: 10.1016/j.immuni.2016.02.010 26948373 10.1016/j.immuni.2016.02.010 PMC4794380

