# Supplementary figures and images for: Transiently antigen primed B cells can generate multiple subsets of memory cells
Source: PLoS One. 2017 Aug 29;12(8):e0183877. doi: 10.1371/journal.pone.0183877 (PMC5574538; doi:10.1371/journal.pone.0183877)

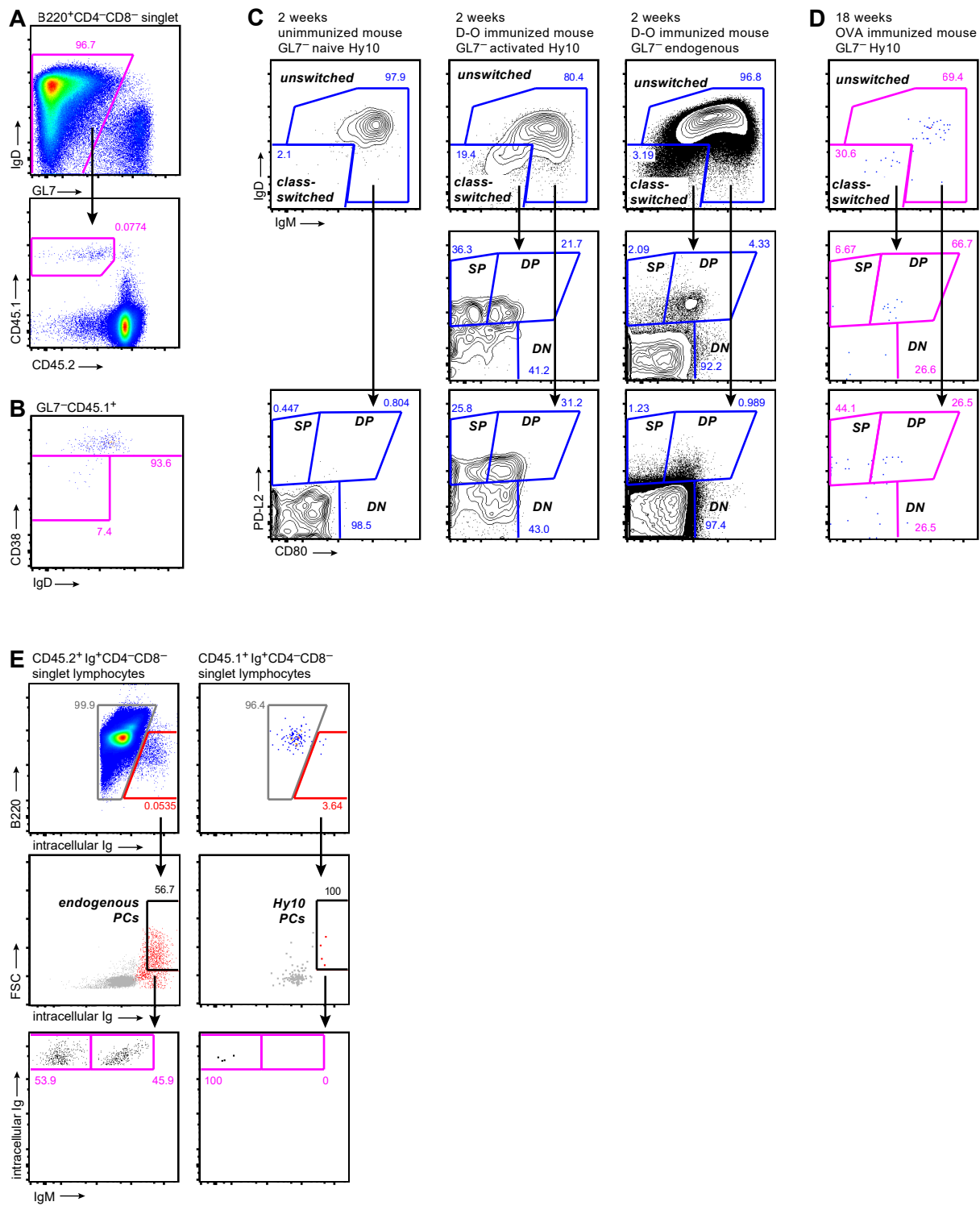

Supplement: S1 Fig — A, Hy10 memory B cell gating strategy. Representative of n = 2–4 experiments with 4–12 mice per condition. 2 week timepoint shown from dLNs. B, CD38 staining of GL7− Hy10 cells in dLNs. Representative of n = 2 independent experiments with 4–6 mice at 2 and 18 weeks after transfer. 2 week timepoint shown. C, Class-switching and memory subpopulation gating. For recovery of high numbers of Hy10 naïve and memory cells for memory subpopulation gating, 5x10^6^ unpulsed or 1x10^6^ DEL-OVA pulsed Hy10 B cells were transferred to naïve and DEL-OVA immunized recipient mice, respectively. Draining LNs from DEL-OVA immunized and peripheral LNs from unimmunized recipients were analyzed 2 weeks after transfer. GL7− Hy10 (left, middle panels) and endogenous (right panels) B cells were gated as in A. Representative of n = 2 independent experiments with 3–4 mice. D, Example of memory subpopulation gating from 18 week timepoint. Representative of n = 4 independent experiments with 6–12 mice. E, Plasma cell gating strategy. PCs were identified as B220lo intracellular Ighi cells (upper panels, red gates) that were larger and stained more brightly for intracellular Ig than B220+ cells (middle panels, black gates). Ig+B220+ cells (grey gates) shown for comparison. Class switched PCs were defined based on intracellular IgM staining (lower panels). (PDF) [file pone.0183877.s001.pdf]

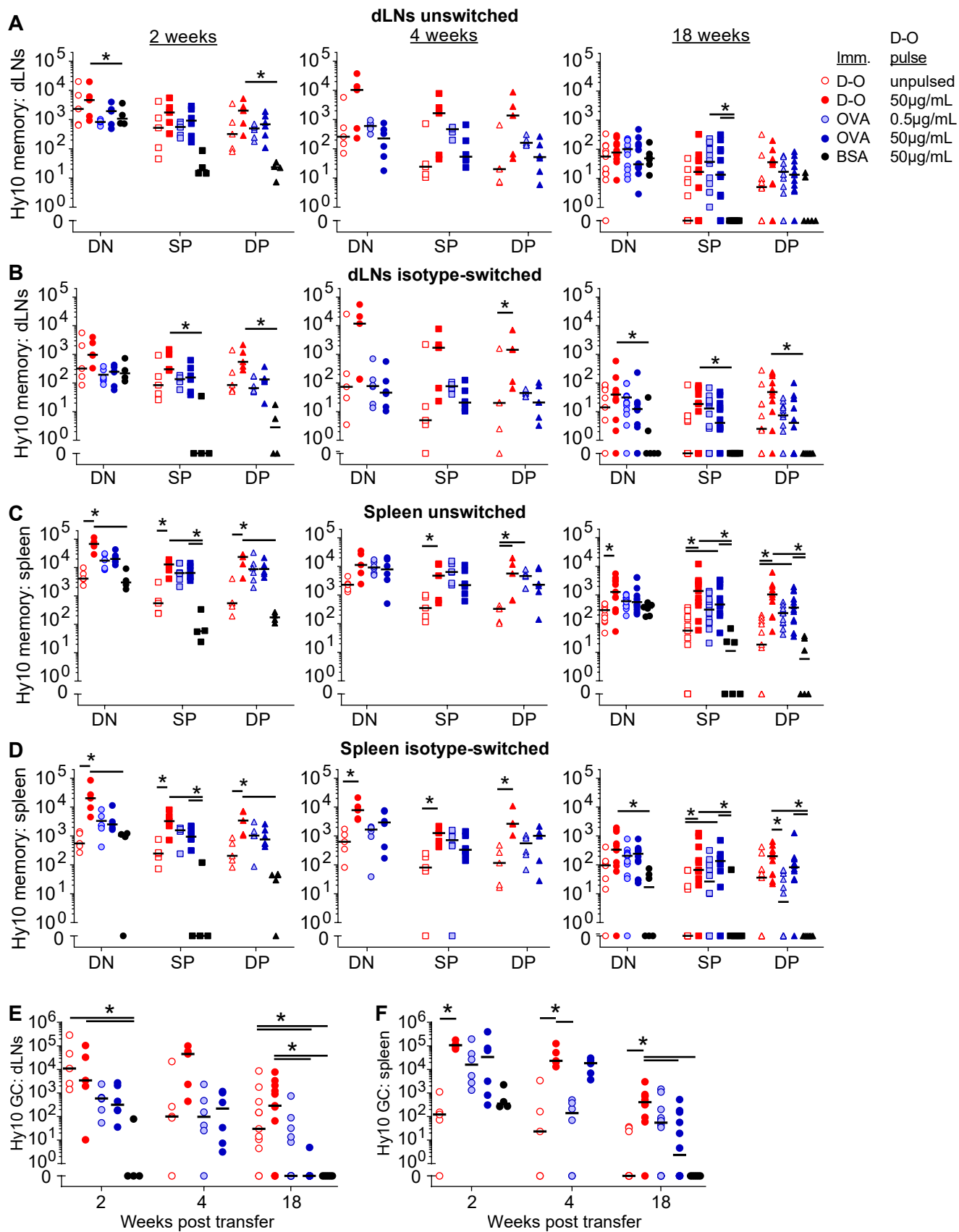

Supplement: S2 Fig — Memory (A–D) and GC (E, F) B cell responses of unpulsed (open symbols) and 50 μg/mL (filled symbols) or 0.5 μg/mL (shaded symbols) DEL-OVA pulsed Hy10 B cells in dLNs (A, B, E) and spleens (C, D, F) of OVA (blue symbols), DEL-OVA (red symbols), and BSA (black symbols) immunized recipient mice 2 weeks (left panels), 4 weeks (middle panels) and 18 weeks (right panels) after transfer. DN, SP, and DP subpopulations gated as in S1A and S1C Fig, and GCs gated as in Fig 2A. All populations shown as total number of cells per dLNs (A, B, E) and spleens (C, D, F). *, P<0.05 (Kruskal-Wallis test with Dunn's post-test between all conditions at each timepoint. Differences between groups not annotated by an asterisk did not reach statistical significance.) (PDF) [file pone.0183877.s002.pdf]
