# Supplementary material for: Transiently antigen primed B cells can generate multiple subsets of memory cells
Source: PLoS One. 2017 Aug 29;12(8):e0183877. doi: 10.1371/journal.pone.0183877 (PMC5574538; doi:10.1371/journal.pone.0183877)
Supplement: S1 Table — (PDF) [file pone.0183877.s003.pdf]

Supplementary Table I. List of antibodies used						
**Target**	**Application**	**Species**	**Fluorochrome(s)**	**Clone**	**Dilution(s)**	**Manufacturer**
B220	FC	Rat	FITC, PE-Cy7, V500	RA3-6B2	50, 100, 50	BD Pharmingen
CD4	FC	Rat	APC-Cy7	RM4-5	100	BioLegend
CD4	FC	Rat	V500	RM4-5	400	BD Biosciences
CD8	FC	Rat	APC-Cy7	53-6.7	100	eBioscience
CD8	FC	Rat	V500	53-6.7	50	BD Biosciences
CD11c	MACS	Arm. Ham.	Biotin	N418	100	BioLegend
CD19	FC	Rat	PE	1D3	100	BD Pharmingen
CD38	FC	Rat	PerCP-eFluor 710	90	200	eBioscience
CD43	MACS	Rat	Biotin	S7	100	BD Biosciences
CD45.1	FC	Mouse	Pacific Blue	A20	100	BioLegend
CD45.2	FC	Mouse	Alexa 700, PerCP-Cy5.5	104	50, 50	BioLegend
CD80	FC	Arm. Ham.	PerCP-Cy5.5	16-10A1	100	BioLegend
CD95	FC	Arm. Ham.	PE-Cy7	Jo2	100	BD Pharmingen
CD138	FC	Rat	biotin	281-2	100	BD Pharmingen
CD273	FC	Rat	APC	TY25	100	BioLegend
GL-7	FC	Rat	Biotin	GL-7	100	eBioscience
IgD	FC	Rat	APC-Cy7	11-26c.2a	100	BioLegend
IgH+L	FC	Goat	Alexa 647	Polyclonal	100	Southern Biotech
IgM	FC	Goat	FITC	Polyclonal	100	Southern Biotech
IgM^a	FC	Mouse	PE	DS-1	200	BD Pharmingen
IgM^b	FC	Mouse	PE	AF6-78	200	BD Pharmingen
Streptavidin	FC	n/a	Qdot 605	n/a	200	Life Technologies
**Legend**						
FC – Flow Cytometry						
MACS – AutoMACS purification						
